# Retinal imaging in animal models: Searching for biomarkers of neurodegeneration

**DOI:** 10.3389/fopht.2023.1156605

**Published:** 2023-04-06

**Authors:** Ana Batista, Pedro Guimarães, Pedro Serranho, Ana Nunes, João Martins, Paula I. Moreira, António Francisco Ambrósio, Miguel Morgado, Miguel Castelo-Branco, Rui Bernardes

**Affiliations:** ^1^ University of Coimbra, Coimbra Institute for Biomedical Imaging and Translational Research (CIBIT), Institute for Nuclear Sciences Applied to Health (ICNAS), Coimbra, Portugal; ^2^ Universidade Aberta, Department of Sciences and Technology, Lisbon, Portugal; ^3^ University of Coimbra, Coimbra Institute for Clinical and Biomedical Research (iCBR), Faculty of Medicine (FMUC), Coimbra, Portugal; ^4^ University of Coimbra, Center for Innovative Biomedicine and Biotechnology (CIBB), Coimbra, Portugal; ^5^ University of Coimbra, Clinical Academic Center of Coimbra (CACC), Faculty of Medicine (FMUC), Coimbra, Portugal; ^6^ University of Coimbra, Center for Neuroscience and Cell Biology (CNC), Coimbra, Portugal; ^7^ University of Coimbra, Department of Physics, Faculty of Sciences and Technology (FCTUC), Coimbra, Portugal

**Keywords:** Alzheimer’s disease, aging, optical coherence tomography, animal models, retinal thickness, texture analysis, deep learning

## Abstract

There is a pressing need for novel diagnostic and progression biomarkers of neurodegeneration. However, the inability to determine disease duration and stage in patients with Alzheimer’s disease (AD) hinders their discovery. Because animal models of disease allow us to circumvent some of these limitations, they have proven to be of paramount importance in clinical research. Due to the clear optics of the eye, the retina combined with optical coherence tomography (OCT) offers the perfect opportunity to image neurodegeneration in the retina *in vivo*, non-invasively, directly, quickly, and inexpensively. Based on these premises, our group has worked towards uncovering neurodegeneration-associated changes in the retina of the triple-transgenic mouse model of familial AD (3×Tg-AD). In this work, we present an overview of our work on this topic. We report on thickness variations of the retina and retinal layers/layer aggregates caused by healthy aging and AD-like conditions and discuss the implications of focusing research efforts solely on retinal thickness. We explore what other information is embedded in the OCT data, extracted based on texture analysis and deep-learning approaches, to further identify biomarkers that could be used for early detection and diagnosis. We were able to detect changes in the retina of the animal model of AD as early as 1 month of age. We also discuss our work to develop an optical coherence elastography system to measure retinal elasticity, which can be used in conjunction with conventional OCT. Finally, we discuss the potential application of these technologies in human patients and the steps needed to make OCT a helpful screening tool for the detection of neurodegeneration.

## 1 Introduction

Over a third of all Europeans suffer from some form of central nervous system (CNS) disease (1). With improved care and life expectancy, the already worrying numbers associated with neurodegeneration are expected to climb even further. The soaring prevalence and economic burden of neurodegeneration pose a significant and mounting threat to every governmental body in the world.

There is a need for novel diagnostic and progression biomarkers for neurodegenerative disorders, such as Alzheimer’s Disease (AD) or Parkinson’s Disease. Despite some advances, the accurate and timely diagnosis of these diseases is still lacking, with studies reporting substandard misdiagnosis rates and initial stages typically remaining undiagnosed (2–5). The eye is a readily available and inexpensive window into human health. The retina is part of the CNS and is the perfect target for *in vivo*, *in situ*, and non-invasive neuropathology diagnosis.

Optical coherence tomography (OCT) is now a standard imaging modality in daily clinical practice. First demonstrated in 1991, it is a non-contact imaging modality based on interferometry. It was first used to image the human retina *in vivo* in 1993 (6). Since then, OCT has revolutionized retinal imaging. OCT systems are fast, non-invasive, inexpensive, and readily available. This starkly contrasts with typical neuroimaging modalities and opens up the possibility of screening. Embedded in the OCT signal there is a wealth of information that can be used to evaluate the retina. The underlying rationale is the sensitivity of OCT to subtle refractive index changes and the amount of data collected from the ocular fundus. Minor differences at the molecular level cannot be individualized, but they still affect the statistics of the global data. Amyloid-beta has been demonstrated in and around melanopsin retinal ganglion cells in AD patients (7). Furthermore, hyperphosphorylated tau is present in mice’s innermost layers of the retina (8). These and other neurodegenerative-related changes lead to differences in the refractive index of the retina and its components that can be detected in OCT scans.

Animal models play a key role in the search for novel diagnostic, progression, and treatment response biomarkers. In the real world, neurodegenerative diseases can remain unsuspected for more than 10 years before a tentative diagnosis is made (9–11). This results in the loss of a pivotal time window (early diagnosis) in which novel disease-modifying therapies could intervene. Furthermore, disease staging is largely unknown, and the assessment of disease progression is qualitative and difficult to quantify. This dramatically hinders the search for progression and treatment response biomarkers. Longitudinal studies require long-term commitment and are cumbersome for subjects. Differences in basic characteristics or lifestyle are difficult to match between populations. Availability of subjects is also a significant limitation. None of these issues extend to animal models of disease. Disease onset and duration are more readily known, and the time scale is compressed, facilitating longitudinal studies. Multiple imaging sessions over months are feasible, and population matching is guaranteed *a priori*.

In this manuscript, we present an overview of our efforts to uncover the missing links between signs in the retina and neurodegeneration using an animal model of AD and age-matched controls. We show the evolution of retinal layer thickness in both groups and discuss the drawbacks of focusing research efforts on retinal thickness alone. We show that retinal aging is altered in the triple-transgenic mouse model of familial AD (3×Tg-AD) and discuss the implications. Using metrics that describe image patterns, we show that changes are detectable as early as 1 month of age. In previous works, we have shown that these changes are widespread and not specific to a single retinal layer. We also discuss the development of an optical coherence elastography (OCE) system that can be used in conjunction with traditional OCT to obtain information on the elastic properties of the retina. Finally, we discuss the possibilities of translating the knowledge gained to humans and the efforts needed to make ocular imaging the screening tool required to deal with neurodegeneration.

## 2 Materials and methods

### 2.1 Ethics statement

All the studies were approved by the Animal Welfare Committee of the Coimbra Institute for Clinical and Biomedical Research (iCBR), Faculty of Medicine, University of Coimbra. All procedures were conducted in accordance with the guidelines for animal use by the Association for Research in Vision and Ophthalmology, which align with the European Community Directive Guidelines for the care and use of nonhuman animals for scientific purposes (2010/63/EU), which have been implemented into Portuguese law in 2013 (DL113/2013).

### 2.2 Data

In total, 57 wild type (WT) C57BL6/129S and 57 triple transgenic (3×Tg-AD) age-matched male mice were used. The 3×Tg-AD mouse carries three mutant genes, namely genes encoding human beta-amyloid precursor protein (APPswe), presenilin-1 (M146V), and microtubule-associated protein tau (P301L), and recapitulates both amyloid and tau pathologies (12). Observed progression timing and localization were shown to mimic human observations. For each mouse, OCT volumes were acquired from both eyes at the ages of 1, 2, 3, 4, 8, 12, and 16 months. Animals were included when available over approximately 8 months. Subpar OCT volumes were excluded from the analysis.

Between acquisitions, animals were maintained in an animal house facility at the iCBR, Faculty of Medicine, University of Coimbra. Animals were kept at controlled temperature and luminosity (12-h light/dark) and with free access to food and water. **Table 1** shows the mean animal weights (and standard deviations) for both groups throughout the entire duration of the experimental setup. Animal weight increased significantly with age for both groups (repeated measures ANOVA: p<0.001 for both groups). No statistical differences were observed between animal groups aged up to 4 months. In mice older than 8 months, WT mice had a significantly higher weight than 3×Tg-AD mice. Statistical differences between the groups were assessed using the independent samples t-test after confirming normality using the Kolmogorov-Smirnov normality test.

**Table 1:** Weight of wild type (WT) mice and the triple transgenic model of Alzheimer’s Disease (3xTg-AD).

Age (Months)	1	2	3	4	8	12	16
WT (g)	14.82	22.63	25.25	26.95	32.69*	35.04*	36.98*
	(2.62)	(1.68)	(1.91)	(2.09)	(3.53)	(3.46)	(4.09)
3×Tg-AD (g)	14.90	22.24	25.60	27.49	30.41	31.66	32.91
	(2.64)	(2.32)	(2.24)	(2.04)	(2.84)	(3.48)	(3.69)
p-values	0.87	0.32	0.40	0.18	6.00×10^-4^	8.61×10^-6^	2.53×10^-6^

Average (standard deviation) values per age and group are shown. Statistical differences between the two groups were assessed with the independent samples t-test and are indicated by * (p < 0.001). The resulting p-values are shown.

Before OCT image acquisition, animals were anesthetized using a mixture of 80 mg/kg ketamine (Nimatek; Dechra) and 5 mg/kg xylazine (Sedaxylan; Dechra). Additionally, oxibuprocaine (Anestocil; Edol) was used as a local anesthetic. Pupil dilation was achieved with 0.5% tropicamide (Tropicil; Edol) and 2.5% phenylephrine (Davinefrina; Dávi) solution. Eye hydration was maintained during acquisition using 1% carmellose drops (Celluvisc; Allergan).

### 2.3 OCT Imaging

OCT volumes were acquired using a Micron IV OCT System (Phoenix Technology Group, Pleasanton, CA, USA). This system employs a superluminescent diode with a bandwidth of 160 nm centered at 830 nm. The imaging depth of the system is 1.4 mm, with an axial resolution of 3 μm. Each volume is composed of 512 B-scans, with each B-scan having 512 A-scans composed of 1024 discrete samples in depth. All B-scans were saved as non-compressed TIFF image files. For consistency, all OCT volumes were acquired by a single operator in a set retinal region. Using the optic disc as a landmark, the imaged retinal region was horizontally aligned with it and positioned directly above it.

### 2.4 Retinal layer segmentation

**Figure 1:**
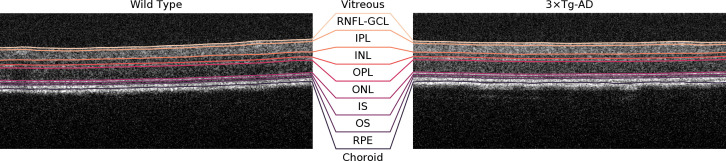
Representative B-scans (1 month old) of a wild type mouse (left) and a triple transgenic mouse model of Alzheimer’s Disease (3xTg-AD) (right) with overlayed segmentations. RNFL-GCL – retinal nerve fiber layer and ganglion cell layer complex; IPL – inner plexiform layer; INL – inner nuclear layer; OPL – outer plexiform layer; ONL – outer nuclear layer; IS – photoreceptors inner segment; OS – photoreceptors outer segment; RPE – retinal pigmented epithelium.

A deep learning-based approach was used to segment the retinal nerve fiber layer and ganglion cell layer complex (RNFL-GCL), inner plexiform layer (IPL), inner nuclear layer (INL), outer plexiform layer (OPL), outer nuclear layer (ONL), photoreceptors inner segment (IS), photoreceptors outer segment (OS), and retinal pigmented epithelium (RPE) (13). Although the RNFL-GCL complex is a layer aggregate and not a single layer, it will herein be defined as so for simplicity. Briefly, the neural network used consists of a convolutional neural network (CNN) with a U-type architecture (14) with shortcut connections between the encoding and decoding paths. The encoding path also uses residual blocks that contain skip-through connections, *i.e.*, connections that skip some layers, which mitigate the problem of exploding/vanishing gradients based on the ResNet architecture (14). The network receives as input a single B-scan and outputs the result of the SoftMax activation as the probability of each pixel belonging to the above-described 8 layers or none of them. Post-processing is applied to define the interfaces between two adjacent layers. The network was trained on manually corrected ground truth. Corrections were performed by two experienced graders. Volumetric segmentation is achieved by combining the 512 segmented B-scans. Representative B-scans and achieved segmentation are shown in **Figure 1**.

### 2.5 Retinal thickness

Retinal thickness maps were computed from the volumetric segmented OCT data. The thickness of each retinal layer, as well as the total retinal thickness (TRT), were computed as the average distance in μm between the respective segmented boundaries. Average thickness values per volume were computed as the average of the 512 × 512 values (1 measurement per A-Scan) minus excluded A-Scans due to segmentation quality. The quality of the segmentation at each position was evaluated before computation. Three exclusion criteria were considered based on data quality, segmentation (boundary) consistency, and distribution of thickness measurements to guarantee the overall quality. The unfulfillment of one or more of these criteria resulted in the exclusion of the individual thickness values from the analysis and average computation. Additionally, we did not consider volumes from which more than 10% of the total thickness measurements were excluded.

### 2.6 Fundus images

**Figure 2:**
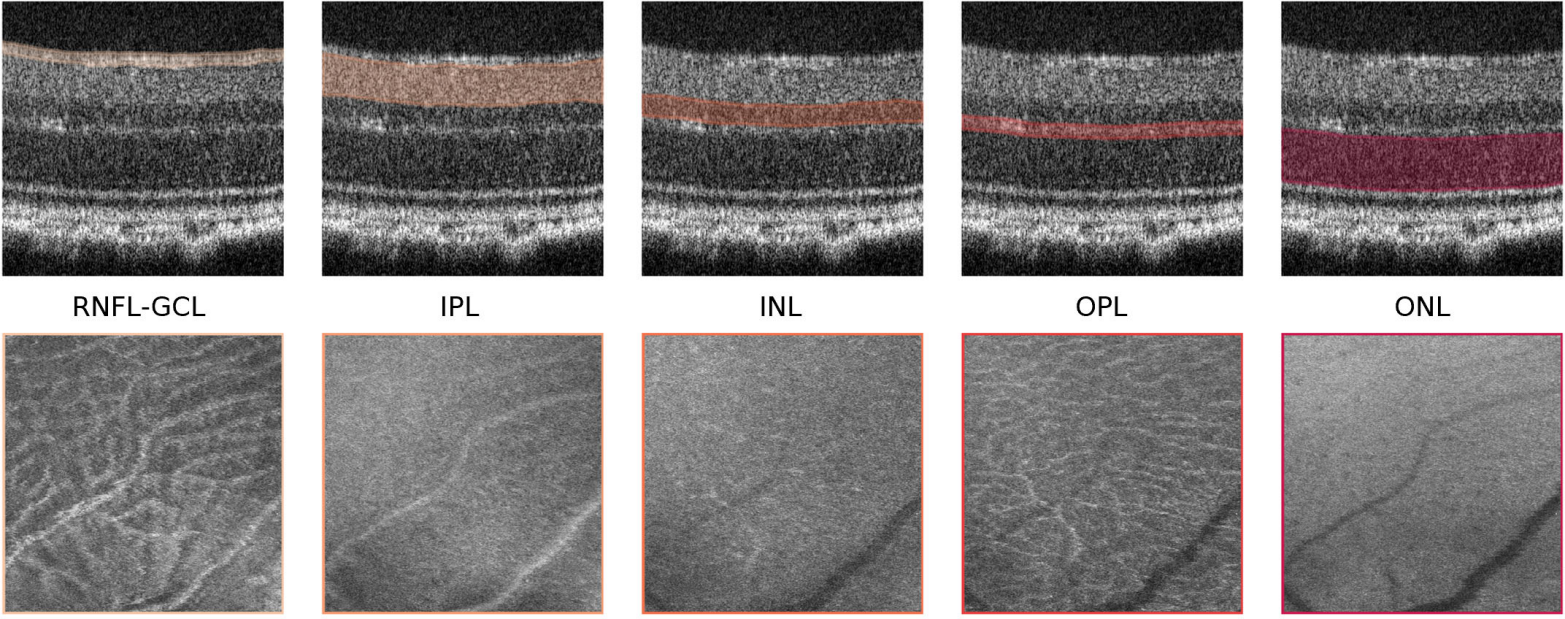
Mean-value fundus (MVF) images. Example of MVF images (bottom) computed from the retinal nerve fiber layer and ganglion cell layer complex (RNFL-GCL), and the inner plexiform layer (IPL), inner nuclear layer (INL), outer plexiform layer (OPL), and outer nuclear layer (ONL). On top, the respective layers used for MVF computation are highlighted.

A mean-value fundus (MVF) image (15) was computed as the average of the A-scan values between the boundaries of the layers for each of the segmented RNFL-GCL complex, the IPL, INL, OPL, and ONL layers. We limited the MVF images to the neuroretina, i.e., retinal layers that are analogous to those found in the brain. These images allow us to project the 3D information of each layer onto a 2D image, similar to retinal fundus photography (**Figure 2**). The differentiating aspect comes from limiting this to specific layers.

### 2.7 Texture features

Texture is the characteristic that describes the spatial distribution of pixel intensity values. The Gray-Level Co-occurrence Matrix (GLCM) is a popular method for texture-related feature extraction (16). For local feature extraction, we applied GLCM to squared blocks of 20 × 20 pixels, cropped from the MVF images. In total, 24 × 24 blocks per image were used, focusing on the central region of the fundus. GLCM was computed at four angles (0, 45, 90, and 135°) using a scale of one pixel. Symmetry was accounted for, i.e., 180° apart angles are considered the same. For each resulting GLCM, 20 features were computed, namely: the Inverse Difference Moment/Energy, Contrast/Inertia, Correlation, Angular Second Moment/Uniformity, Sum Average, Sum of Squares, Sum Variance, Sum Entropy, Difference Variance, Difference Entropy, Information Measure of Correlation 1 (IMC1), Information Measure of Correlation 2 (IMC2), and Entropy were computed as described in (16); Autocorrelation and Maximum Probability as described in (17); Cluster Prominence and Cluster Shade as described in (18); Inverse Difference Normalized (INN) and Inverse Difference Moment Normalized (IDN) as described in (19); and Dissimilarity as described in (20). Across the four directions, only the maximum feature value was considered. Blocks were aggregated into quadrants by defining each quadrant feature’s value as the average of 12 × 12 blocks. The feature extraction process was repeated five times for the MVF images computed from each of the segmented RNFL-GCL complex, the IPL, INL, OPL, and ONL layers. This results in a total of 80 features (20 features × 4 quadrants) per layer (5) per acquisition time point (7), which were then subjected to feature selection (described in Statistical Analysis). Further details on the feature extraction process can be found in (21).

### 2.8 Age modeling

Accelerated aging or a positive brain-age gap in neural tissue has been associated with AD in adult humans. Age-related changes, such as neural activity and functional connectivity impairments, have been shown to be more pronounced in AD (22, 23).

We trained and tuned two convolutional neural networks based on the DenseNet architecture (24) to predict biological age from OCT data: one using only B-scans from WTs, and the other using only B-scans from 3×Tg-AD mice. The network receives as input a single B-scan and outputs an age prediction. Both models were used to predict age in an independent dataset, which included B-scans from both WT and 3×Tg-AD mice. Further details on the network and protocol can be found in (25).

### 2.9 Classification

A convolutional neural network based on the Inception-v3 network (26) trained on the ImageNet dataset (27) was applied to classify images as belonging to the WT or 3×Tg-AD mouse group, regardless of the age. The network receives as input an MVF image and outputs a group prediction.

Mice were first split into train/tuning and test sets (80/20% of mice). Then the train/tuning set was further divided into the train (75%) and tunning (25%) sets. Our goal was to answer the following three questions: (1) Can a neural network be trained to consistently discriminate between the two groups over an extended period? (2) Can it be trained to recognize differences between these groups at times outside the training period? (3) Is the information layer-specific or widespread? Thus, the network was trained exclusively with data from mice at the ages of 3, 4, and 8 months. OCT scans from 1-, 2-, and 12-month-old mice were left out exclusively for testing. This allows us to understand the ability of the neural network to classify mice that are either younger or older than those in training. Further details of the network and protocol can be found in (28).

To infer how widespread in the retina the information is, training, tuning, and testing were repeated five times as an independent CNN model was created for MVF images computed from each of the segmented RNFL-GCL complex, the IPL, INL, OPL, and ONL layers.

### 2.10 Statistical analysis

We used the Kolmogorov-Smirnov normality test to assess the normal distribution of the data at a significance level of 10%. Statistical differences between groups were evaluated using the independent samples t-test when normality was not rejected, and the non-parametric alternative Mann-Whitney U-test, when at least one of the groups did not follow a normal distribution. Significance levels of 5%, 1%, and 0.1% were considered. After comparing the texture between the two groups, the Pearson correlation, if features were distributed normally, or if not, Spearman correlation, was computed for feature pairs. For each comparison, if two features were correlated (p<0.05), only the feature with the lowest p-value (intergroup comparison) was considered. The Bonferroni correction was performed to correct for multiple comparisons.

## 3 Results

### 3.1 Retinal thinning

**Figure 3:**
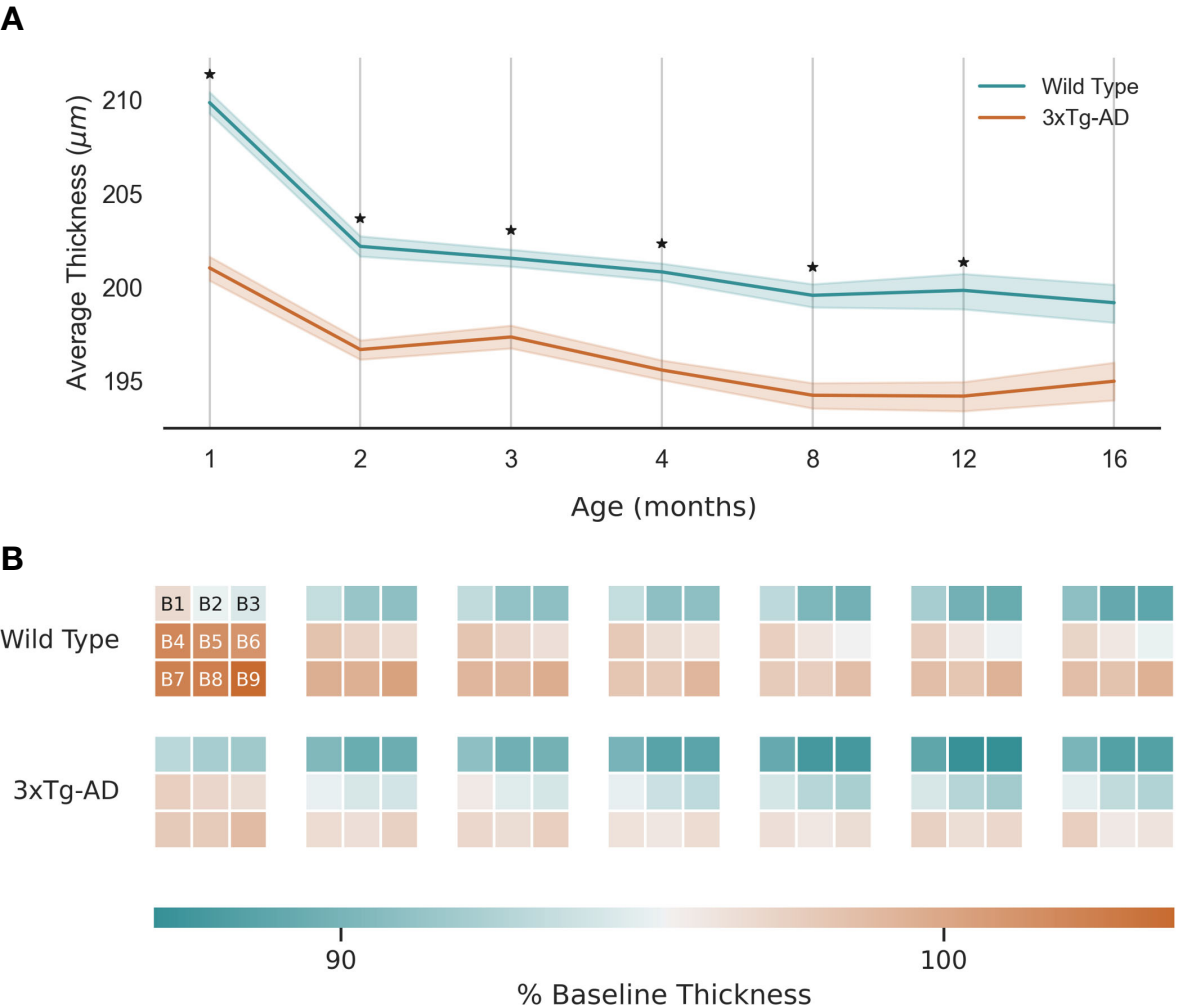
Retinal thickness longitudinal variation: **(A)** - Average thickness and standard deviation (line and filled area, respectively) over 16 months for wild type and the triple transgenic (3xTg-AD) mice. **(B)** - Average thickness variation per region (B1-9, as shown). Thickness presented as % of baseline thickness, defined as the average retinal thickness of wild type mice aged 1 month. Color scale as indicated by the color bar. ★ p<0.001.

TRT for WT and 3×Tg-AD mice is shown in **Figure 3A**, defined as the distance between the upper boundary of the RNFL-GCL complex and the lower boundary of the RPE. Data from both eyes were included. As shown, TRT decreases with age for both groups. The retinas of WT mice were significantly thicker than those of transgenic mice at all ages (p<0.01, **Figure 3A**). Notably, at the first time point, the TRT of 3×Tg-AD mice is already significantly thinner. Only at the age of 16 months were the differences not statistically significant (p>0.05). When the thickness values of the left and right eyes are addressed separately, statistical differences between WT and 3×Tg-AD TRT were found at all ages (data not shown). Thinning appears to occur at a faster rate for WT mice between 1 and 2 months of age. Similar behavior is observed in 3×Tg-AD mice, although at a slower pace.

**Figure 3B** shows the local variation by dividing the retina into 9 equally sized regions of interest (B1-9, covering 510×510 A-scans centrally cropped from the OCT volumes). As expected, TRT decreases with distance from the optic nerve. Although 3×Tg-AD retinas are thinner overall, there is no clear group-specific thinning pattern.

**Figure 4:**
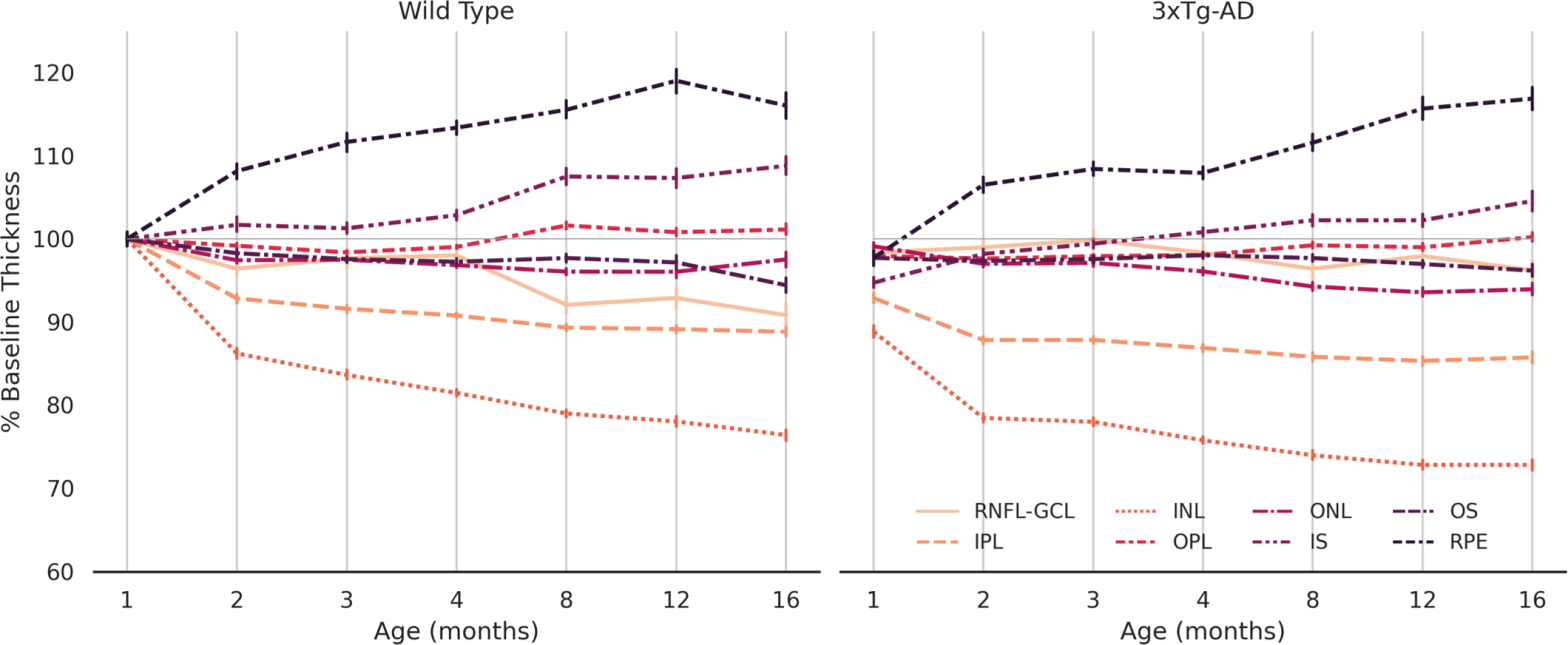
Normalized average thickness. Average thickness values over 16 months for wild type and the triple transgenic (3xTg-AD) mice (left and right, respectively) discriminated per retinal layer (RNFL-GCL complex and IPL, INL, OPL, ONL, IS, OS, and RPE layers). Thickness presented as % of baseline, defined as the average retinal thickness per retinal layer of wild type mice aged 1 month. Error bars show standard deviation. RNFL-GCL – retinal nerve fiber layer and ganglion cell layer complex; IPL – inner plexiform layer; INL – inner nuclear layer; OPL – outer plexiform layer; ONL – outer nuclear layer; IS – photoreceptors inner segment; OS – photoreceptors outer segment; RPE – retinal pigmented epithelium.

**Figure 4** discriminates variation in retinal thickness per segmented layer. In both groups, the IPL and INL layers were the most thinned. On the other hand, both the RPE and IS layers thicken over time. Between the two groups, the behavior of RNFL-GCL complex seems to differentiate the most. A thorough analysis will be the subject of future work.

### 3.2 Texture differences

**Figure 5:**
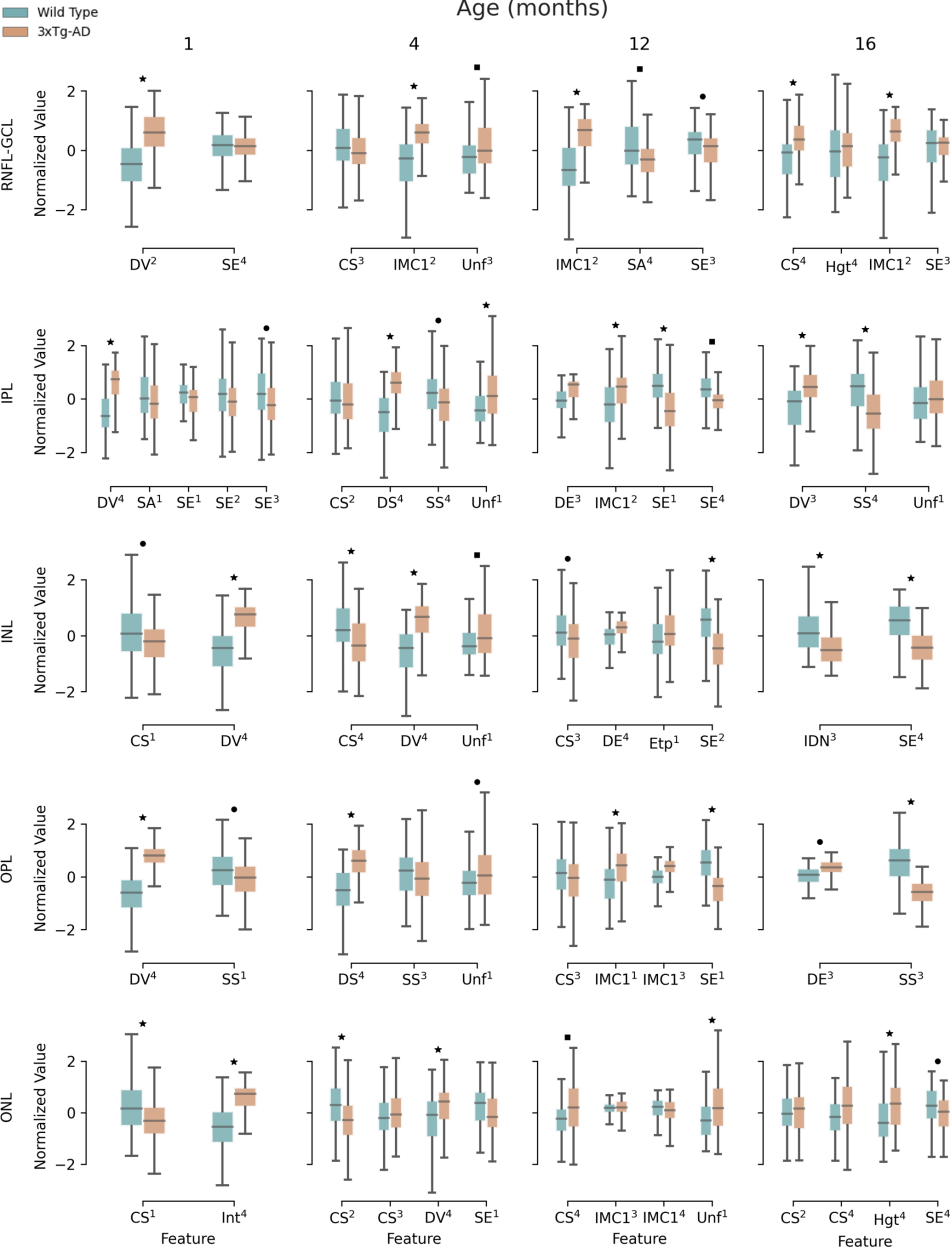
Retina texture. Boxplots for extracted textural features for 1-, 4-, 12-, and 16-month-old mice, for wild type and the triple transgenic (3xTg-AD) mice discriminated per retinal layer (RNFL-GCL complex and IPL, INL, OPL, ONL, IS, OS, and RPE layers). Median, first, and third quartiles are represented. Whiskers at each quartile plus 1.5 times the interquartile range. All features were normalized to have zero mean and unit standard deviation. Feature name and quadrant (superscript) as indicated. Correlated features (p<0.05) per layer and time point were excluded. CS – Cluster Shade; DE – Difference of Entropy; DS – Dissimilarity; DV – Difference of Variance; Etp – Entropy; Hgt – Homogeneity; IDN – Inverse Difference Moment Normalized; IMC1 – Information Measure of Correlation 1; SA – Sum Average; SE – Sum of Entropy; SS – Sum of Squares; Unf – Uniformity; RNFL-GCL – retinal nerve fiber layer and ganglion cell layer complex; IPL – inner plexiform layer; INL – inner nuclear layer; OPL – outer plexiform layer; ONL – outer nuclear layer. ▪ p<0.05; ● p<0.01; ★ p<0.001. These limits were corrected for multiple comparisons using the Bonferroni method.

**Figure 5** shows the results of the textural comparison between WT and 3×Tg-AD mice. For the sake of convenience, the results portrayed are limited to 1-, 4-, 12-, and 16-month-old mice. Results for the remaining time points can be found in the **Supplementary Material**. As expected, there were many correlations between computed features. After feature selection, only a handful of features per time point and layer remain. Significant differences between WT and 3×Tg-AD mice can be found from month 1. At least one feature is significantly different (p<0.001) for each time point and layer. The widespread statistically significant differences throughout the layers and across time points highlight the considerable influence of the three gene mutations that characterize the mouse model of familial AD.

### 3.3 Age-gap

**Figure 6:**
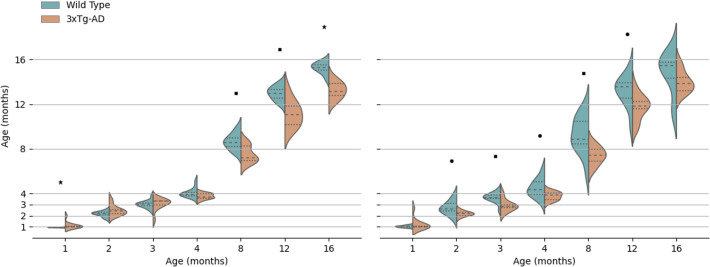
Kernel density estimate of predicted retinal age per class (wild type and the triple transgenic mice, WT, and 3×Tg-AD, respectively) for each age. On the left is the prediction of the network trained exclusively on WT mice, and on the right is the network trained exclusively on 3×Tg-AD mice. The median (dashed), and first and third quartiles (dotted) are shown. Figure adapted from (25). ▪ p<0.05; ● p<0.01; ★ p<0.001.

Two models were created by training a convolutional neural network to predict the age of WT and 3×Tg-AD mice from OCT B-scans. We then used an independent dataset to test the two resulting models. Because each model was trained exclusively on one group (WT or 3×Tg-AD), we eliminated training bias. **Figure 6** shows the kernel density age estimates for each acquisition time-point, separated per class for both models. Overall, regardless of the training group, it was possible to predict the age of mice with a reasonable degree of accuracy. As predicted by the models, retinal aging differs between the two classes. After month 4, WT retinas are consistently predicted to be older than 3×Tg-AD retinas. This is consistent between the two models, substantiating obtained results. These results have been further described by (25).

### 3.4 Deep learning opens the door to early detection

We used a neural network to classify computed fundus images from OCT scans of the retinas of WT and 3×Tg-AD mice. **Table 2** discriminates misclassification rates per layer and time-point for the classification of young (1 and 2 months old) and older (12 months old) mice using scans from 3-, 4-, and 8-month-old mice to train and tune the network. In general, the performance of the networks is particularly good. It demonstrates that the network can extrapolate to ages not included in the training set. As shown, most misclassifications occur for 1 month old retinas. Also, the misclassification rate increases at deeper layers of the retina, especially when both groups are considered. The number of misclassifications increases from the RNFL-GCL complex to the ONL. 12-month-old mice have the lowest misclassification rate.

**Table 2:** Percentage of classification errors for wild type (WT) and the triple transgenic mouse model of Alzheimer’s disease (3×Tg-AD) discriminated by age.

	Age (Months)	1	2	12	All Ages
**WT**	RNFL-GCL	5.3%	16.7%	13.3%	11.5%
	IPL	10.5%	16.7%	20.0%	15.4%
	INL	21.1%	11.1%	20.0%	17.3%
	OPL	5.3%	11.1%	6.7%	7.7%
	ONL	10.5%	33.3%	20.0%	21.2%
	All Layers	10.5%	17.8%	16.0%	14.6%
**3×Tg – AD**	RNFL-GCL	15.8%	19.0%	11.8%	15.8%
	IPL	57.9%	14.3%	5.9%	26.3%
	INL	47.4%	14.3%	17.6%	26.3%
	OPL	78.9%	19.0%	23.5%	40.4%
	ONL	63.2%	14.3%	11.8%	29.8%
	All Layers	52.6%	16.2%	14.1%	27.7%

RNFL-GCL, retinal nerve fiber layer and ganglion cell layer complex; IPL, inner plexiform layer; INL, inner nuclear layer; OPL, outer plexiform layer; ONL, outer nuclear layer. Totals per time point and group are presented.

**Figure 7:**
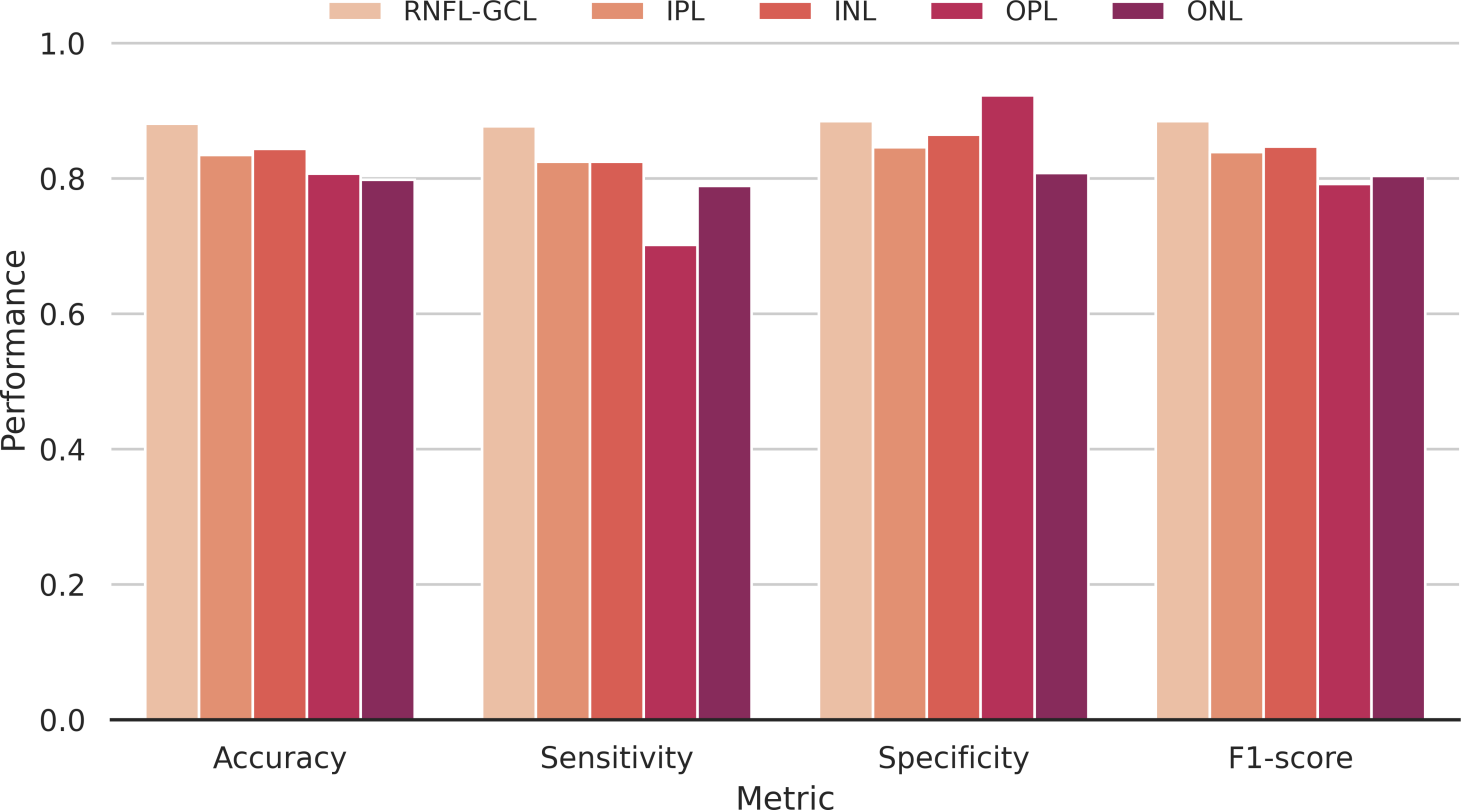
Classification performance. Accuracy, sensitivity, specificity, and F1-score metrics discriminated per segmented layer used to compute the input images. RNFL-GCL – retinal nerve fiber layer and ganglion cell layer complex; IPL – inner plexiform layer; INL – inner nuclear layer; OPL – outer plexiform layer; ONL – outer nuclear layer.

The accuracy, sensitivity, specificity, and F1-score metrics were also used to evaluate the model’s performance per layer (**Figure 7**). Overall, the accuracy is high (>79.8%), regardless of the retinal layer, showing the discriminative power of the networks to distinguish between WT and transgenic mice. As the training group incorporates retinas of distinct ages, the network is forced to learn common features across a time window, which it can then apply to younger and older mice outside its training range. These results, among others, have been further described in (28).

## 4 Discussion

Animal models of disease are of paramount importance in clinical research. They allow us to circumvent major limitations of patient-based studies, such as the uncertainty of disease onset and duration, and the inability to assess changes in the early stages of the disease. With these models, disease duration and staging are more readily known, age-matching is granted *a priori*, and longitudinal studies are easier to conduct. For these reasons, animal models are often chosen as surrogates to investigate pathology-induced changes in the retina, including those related to neurodegeneration, even acknowledging the significant difference between animal models and humans.

The retina, which shares a common origin with the brain during embryonic development, is part of the CNS and has many similarities in terms of its anatomy, vascularization, and immunology, among others. The clear optics of the eye allows for direct observation, providing a valuable opportunity to directly image the effects of AD in the retina. Unlike other methods used to diagnose and monitor neurodegenerative disorders, OCT is non-invasive, fast, inexpensive, and readily available.

We continuously monitored two groups of mice, WT and 3×Tg-AD mice, until 16 months of age. Animals were frequently imaged with OCT, as we brought retinal changes induced by healthy aging and familial AD-associated genes to light. For the first time, the retinas of mice have been meticulously imaged from an early age (1 month old), revealing changes at the very beginning of these animals’ lives. We have investigated readily quantifiable alterations, such as retinal thickness, but also information embedded in the OCT data that could be used for early detection and diagnosis.

We have trained and tuned a neural network model capable of segmenting retinal layers from the OCT of mice. This allowed us to assess the TRT and the thickness of individual layers or aggregates. As expected, both WT and 3×Tg-AD mice show a progressive decrease in total retinal thickness with age. However, we found that not all retinal layers follow this trend. The end-result retinal thinning derives from a layer thickening and thinning balance. We observed a decrease in the thickness of the IPL and INL over time, combined with the thickening of the IS and RPE. The remaining layers have minor to moderate thickness variations.

We observed significantly lower total retinal thickness values in 3×Tg-AD mice compared to age-matched WT animals at 6 out of the 7 ages evaluated. The observed thinning is consistent with the literature for other animal models of AD (APP/PS1 mouse model) (29) and human patients (30). Although RNFL thinning is usually considered a hallmark of AD, in this study we observed RNFL-GCL thinning only in WT mice. In (31), we have already detailed the evolution of retinal thickness in these animal models up to 4 months of age. In the future, we will provide a more thorough analysis up to month 16. We believe that normative retinal thickness data, both layer-specific and for the whole retina, covering the 16 months is an essential comparative reference for future studies.

While verified thickness differences between the two groups can indicate the potential of the retina as a surrogate for neuroimaging, using thickness-based analysis alone has several drawbacks: (1) the potential for error due to changes in segmentation methodology, systems, resolution, scanning protocol, and location, (2) low specificity as retinal thinning is not specific to neurodegeneration and can also be caused by healthy aging or other eye conditions, highly prevalent in the age group most associated with neurodegeneration, and (3) forgoing a wealth of information embedded in the OCT scans.

We computed MVF images, i.e., depth projections, from the RNFL-GCL complex, and INL, IPL, ONL, and OPL layers. These images allowed us to quantify textural differences between WT and 3×Tg-AD mice retinas. The rationale is that minor changes at the molecular level can lead to changes in the tissue refractive index detectable by OCT and translatable into textural differences. We resorted to GLCM, a popular method for texture feature extraction, and computed 80 features per layer and time point. We observed that for every acquisition time point and layer, there were statistically significant differences between the two groups (p<0.001). These indicate that changes are widespread across the retina and persistent in time. The three genes defining the mouse model of familial AD have an enormous phenotypic impact on the retina, which OCT can easily capture. As the retina is part of the CNS, the obtained results suggest that there may be a wide-reaching effect on the brain that can be evaluated by the analysis of the retina.

In the present study, we found statistically significant differences for both retinal thickness and texture at the first acquisition time point (1 month old mice). At this developmental stage, the two groups already presented widespread differences across the retina. This is a limitation of our study, as it was not possible to identify a branching point between WT and 3×Tg-AD. We may speculate that early neurodevelopmental differences exist around or even before birth. For future studies, our results show that the assumption of parity between WT and 3×Tg-AD at early stages may not be correct. This should be taken into account when studying progression.

Age-related changes in the brain, such as neural tissue thinning, neural activity, and functional connectivity impairments, have been associated with AD (22, 23, 32, 33). Brain-predicted age has been reported as a significant predictor of dementia progression (34). We trained and tuned two models to predict mice age from retinal OCT data to understand if similar changes were present in the retina. To eliminate training bias, one model was trained using only WTs’ OCT scans, while the other relied on 3×Tg-AD retinal data. Both models could predict mice age for both WT and transgenic mice. Moreover, our results indicate that the presence of the three mutated genes associated with the familial form of AD impacts retinal aging. Both models consistently predicted ages for WT mice older than 4 months above the ones predicted for 3×Tg-AD mice.

These findings are somewhat unexpected as they appear to contradict what has been observed in the brain, which may partly be explained by a background of early neurodevelopmental changes. Overall, the underlying biological changes leading to age-dependent alterations are complex. Several age-dependent cellular and molecular changes have been previously reported in the retina, such as photoreceptor mislocalization (35), vascular and RPE changes (36), an increase in tissue autofluorescence at the photoreceptor-RPE interface (37), and scattering diversity (38). Nevertheless, these changes are not directly captured by OCT, and a straightforward comparison with our results is not possible. Indeed, what we can infer is that age-dependent neural adaptations that naturally correlate with age are captured by our method. Thus, we can theorize that these healthy adaptations may be altered in transgenic animals. A delay in neural development has been previously described in an AD mouse model (39). Moreover, biological interpretations are further hindered by the complex interactions between inserted mutated genes and the mice’s background. Additional functional and molecular tests are necessary to determine the factors responsible for these findings.

As aforementioned, the onset and staging of AD are difficult to determine. Furthermore, AD typically remains undiagnosed for over 10 years before clinical diagnosis (9–11). Thus, approaches that can classify outside the training domain are paramount if models are to be applied in the real world. Particularly, approaches that can be trained in the late stages and still perform well in the early stages open the door to early detection. An enticing possibility that may lead to novel and effective disease-modifying therapies.

Age modeling alone revealed statistically significant differences between 3×Tg-AD and WT mice aged 8 months or older, even though it was not explicitly trained to distinguish between the two groups. Thus, we trained and tuned a CNN to distinguish between 3×Tg-AD mice retinas and those of age-matched WT animals using data exclusively from mice aged 3, 4, and 8 months. The resulting network was able to distinguish between the two groups when applied to an independent set of fundus images computed from both older (12 months old) and younger (1 and 2 months old) mice retinas. Furthermore, the results have shown that the retinas of transgenic and WT mice can be discriminated based on MVF images of the segmented RNFL-GCL complex, the IPL, INL, OPL, and ONL layers. Observed textural differences support this finding. The effects of the three mutated genes linked to the familial form of AD are not restricted to a particular layer or area of the retina but rather are observed throughout the retina.

The number of errors associated with group prediction was higher for mice aged 1 month, particularly for the correct identification of 3×Tg-AD mice retina. This observation may be related to mice’s CNS development. The mass of mice’s CNS increases from ages 4 to 15 weeks (40). As shown in **Figure 3**, the biggest jump in healthy age-dependent retinal thinning occurs between the first and second month. Thus, it is possible that, at this stage, the retinas of transgenic and WT mice are far removed from the cases the network was exposed to in training.

Evaluation of the elastic properties of the retina may also provide a wealth of information on AD-induced changes. Indeed, the potential of elastography techniques, particularly magnetic resonance elastography (MRE), to detect changes in the brain’s microstructure before volumetric changes or neuronal loss has already been demonstrated (41). Nonetheless, the high cost of MRE hinders its widespread adoption. We are currently developing an OCE system to evaluate changes in retinal elastic properties induced by neurodegeneration in animal models of disease (42). Moreover, we are developing novel approaches to retrieve tissue elastic properties with a higher degree of accuracy, allowing the detection of slight variations (43).

OCE combines an OCT system with a localized applied force to induce tissue displacements. Because they are based on the same underlying system, OCE benefits from some of the advantages of OCT in diagnosing and monitoring neurodegenerative diseases: it is non-invasive and fast. Moreover, it is cost-effective and reachable, since both imaging modalities can be performed in a single examination, and it confers a new level of multimodality that can bring us closer to in-clinic detection of subtle changes in retinal properties in the early stages of neurodegeneration.

In this manuscript, we have highlighted the advantages of using animal models in the study of neurodegeneration. However, there is a significant limitation to this work: there is yet no guarantee that our findings can be translated into clinical applicability. If our findings are realized and verified in humans, OCT could become a powerful screening tool for AD. However, we need prospective studies with real-world human data to understand the feasibility of translation. Furthermore, the “black box” nature of the deep learning approaches used here implies that one cannot infer the reasoning behind each classification decision. It is crucial to ensure that the outcomes of algorithms that inform medical decisions affecting human health are not arbitrary. Patients and clinicians need and deserve a clear-cut explanation of the course of their diagnosis and follow-up. Before the widespread adoption of these methods, we must strive to have more usable and interpretable models.

## Data availability statement

The raw data supporting the conclusions of this article will be made available by the authors upon formal and reasonable request.

## Author contributions

RB and MM accounted for fund raising. RB, PG and PS contributed to the conceptualization and study design. RB and PS contributed to the project administration. JM performed OCT scans. AB and PG performed data analysis. AB, PS and PG contributed to the statistical analysis. AB, PG, PS, AN, JM, PM, AA, MM, MC-B, and RB performed the analysis and interpretation of data. AB, PG, PS, and RB completed an initial review and provided significant edits and additional content before review and approval by the other authors. All authors contributed to the article and approved the submitted version.
