# Supplementary material for: Retinal imaging in animal models: Searching for biomarkers of neurodegeneration
Source: Front Ophthalmol (Lausanne). 2023 Apr 6;3:1156605. doi: 10.3389/fopht.2023.1156605 (PMC11182266; doi:10.3389/fopht.2023.1156605)
Supplement: Supplementary file 1 [file DataSheet_1.pdf]

Supplementary Material – Retinal imaging in animal models: searching for biomarkers of neurodegeneration

Ana Batista[1§], Pedro Guimarães[1§], Pedro Serranho[1,2], Ana Nunes[1], João Martins[1,3,4,5], Paula I. Moreira[4,5,6], António Francisco Ambrósio[3,4,5], Miguel Morgado[1,7], Miguel Castelo-Branco[1,5], Rui Bernardes*[1,5]

1 University of Coimbra, Coimbra Institute for Biomedical Imaging and Translational Research (CIBIT), Institute for Nuclear Sciences Applied to Health (ICNAS), Coimbra, Portugal

2 Universidade Aberta, Department of Sciences and Technology, Lisbon, Portugal

3 University of Coimbra, Coimbra Institute for Clinical and Biomedical Research (iCBR), Faculty of Medicine (FMUC), Coimbra, Portugal

4 University of Coimbra, Center for Innovative Biomedicine and Biotechnology (CIBB), Coimbra, Portugal

5 University of Coimbra, Clinical Academic Center of Coimbra (CACC), Faculty of Medicine (FMUC), Coimbra, Portugal

6 University of Coimbra, Center for Neuroscience and Cell Biology (CNC), Coimbra, Portugal

7 University of Coimbra, Department of Physics, Faculty of Sciences and Technology (FCTUC), Coimbra, Portugal

[§] Authors contributed equally to this work

***Correspondence:**
Rui Bernardes
rmbernardes@fmed.uc.pt

Keywords: Alzheimer's disease, aging, optical coherence tomography, animal models, retinal thickness, texture analysis, deep learning

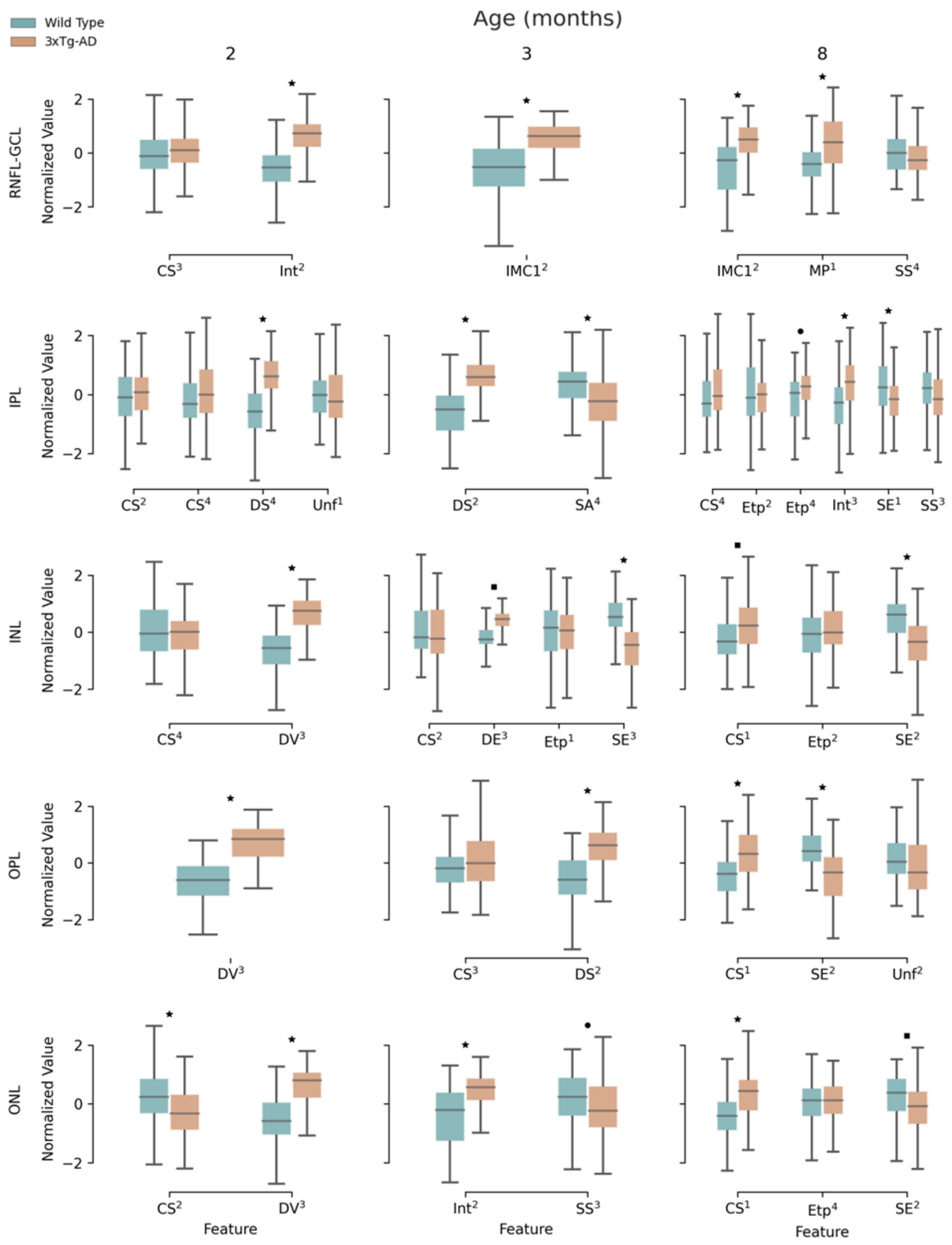

Supplementary Figure 1. Retina texture. Boxplots for extracted textural features for 2-, 3-, and 8-month-old mice, for wild type and the triple transgenic (3xTg-AD) mice discriminated per retinal layer (RNFL-GCL complex and IPL, INL, OPL, ONL, IS, OS, and RPE layers). Median, first, and third quartiles are represented. Whiskers at each quartile plus 1.5 times the interquartile range. All features were normalized to have zero mean and unit standard deviation. Feature name and quadrant (superscript) as indicated. Correlated features ($p<0.05$) per layer and time point were excluded. CS – Cluster Shade; DE – Difference of Entropy; DS – Dissimilarity; DV – Difference of Variance; Etp – Entropy; Hgt – Homogeneity; IDN – Inverse Difference Moment Normalized; IMC1 – Information Measure of Correlation 1; SA – Sum Average; SE – Sum of Entropy; SS – Sum of Squares; Unf – Uniformity; RNFL-GCL – retinal nerve fiber layer and ganglion cell layer complex; IPL – inner plexiform layer; INL – inner nuclear layer; OPL – outer plexiform layer; ONL – outer nuclear layer. ■ $p<0.05$; ● $p<0.01$; ★ $p<0.001$. These limits were corrected for multiple comparisons using the Bonferroni method.
